# A specific combination of dual index adaptors decreases the sensitivity of amplicon sequencing with the Illumina platform

**DOI:** 10.1093/dnares/dsaa017

**Published:** 2020-08-18

**Authors:** Yuu Hirose, Takuhei Shiozaki, Itsuki Hamano, Shizue Yoshihara, Hayato Tokumoto, Toshihiko Eki, Naomi Harada

**Affiliations:** 1 Department of Applied Chemistry and Life Science, Toyohashi University of Technology, Tempaku, Toyohashi, Aichi 441-8580, Japan; 2 Atmosphere and Ocean Research Institute, The University of Tokyo, Kashiwa, Chiba 277-8564, Japan; 3 Department of Biological Sciences, Osaka Prefecture University, Sakai, Osaka 599-8531, Japan; 4 Japan Agency for Marine-Earth Science and Technology, Yokosuka, Kanagawa 237-0061, Japan

**Keywords:** Illumina, dual index adaptor, Nextera, amplicon sequencing

## Abstract

Amplicon sequencing is a powerful approach in microbiome studies as it detects live organisms with high sensitivity. This approach determines the composition of sequence variants of marker genes using high-throughput DNA sequencers. The use of dual index adaptors is the fundamental technique for pooling DNA libraries for Illumina sequencers and is believed not to affect the results. However, here, we observed a decrease of sequence quality in samples containing a specific combination of indexes, namely N704 and S507 in Nextera kits, in multiple runs on the Illumina MiSeq sequencer operated in different facilities. This decrease was also observed when sequencing randomly fragmented DNA of *Escherichia coli* and was not observed when either individual adaptor was used. Each end of the DNA library with this index combination contains a complementary sequence motif, which potentially inhibits proper cluster generation and/or subsequent sequencing. Community analysis of the 16S and 18S rRNA amplicons using QIIME2 revealed significant decreases in α-diversity in the samples containing the N704/S507 index combination, resulting from loss of low-abundance sequence variants during denoising. Our data underscore the importance of quality validation of sequence reads in developing dual index techniques and suggest cautious interpretation of microbiome data containing low-quality sequence reads.

## 1. Introduction

Analysis of the community structure of living organisms is an important aspect of understanding the overall function of an ecosystem. Although isolation and cultivation of organisms is the gold standard for characterization of their physiological, biochemical and genetic properties, most micro-organismal taxa on our planet have yet to be cultured^1^; moreover, the cultivation process often changes the overall structure of the community.^2^ In this regard, culture-independent approaches, such as amplicon sequencing and shotgun metagenomics, have become commonplace with the development of high-throughput DNA sequencers. Amplicon sequencing can reveal the composition of organisms in an ecosystem with a high degree of sensitivity, and therefore this technique is replacing classical techniques such as denaturing gradient gel electrophoresis and Sanger sequencing of cloned DNA.^3^ This approach is superior to shotgun metagenomics with respect to sequencing cost per sample and is effective for the analysis of ecosystems in which phenotypes are tightly associated with taxonomy. Amplicon sequencing has been widely applied to the studies of the human microbiome^4^^,^^5^ as well as other microbiomes of diverse environments on earth.^6–9^

Generally, community analysis using amplicon sequencing is performed with the following steps: (i) preparation of DNA libraries by amplification and multiplexing of target genes, (ii) pooling and sequencing of the libraries using high-throughput sequencers, (iii) denoising and/or clustering of the produced sequences, (iv) taxonomy assignment of the sequences using reference databases and (v) analyses of α-diversity (within-sample diversity) and β-diversity (between-sample diversity). Each step involves several choices. The genes for 16S and 18S rRNAs, which encode the small-subunit rRNAs of prokaryotes and eukaryotes, respectively, are widely used as marker genes. Each rRNA gene contains nine hypervariable regions,^10^^,^^11^ and they are often sequenced together by spanning reads (e.g. regions V3–V4). For sequencing platforms, short read sequencers such as MiSeq (Illumina) and Ion Torrent PGM (Thermo Fisher Scientific) produce highly accurate sequence data with read lengths of 300–500 bp, whereas long-read sequencers such as MinION (Oxford Nanopore) and Sequell (Pacific Biosciences) produce sequence data that are less accurate but have much longer reads, with dozen of kilobase pairs.^3^^,^^12^ Sequencing errors have conventionally been dealt with clustering of sequences into operational taxonomic units (OTUs) based on a distance matrix at a specified threshold (e.g. sequence identity, 97%). Recently, however, sequence errors have been removed based on their quality scores and abundances using the software package dada2,^13^ deblur^14^ or UNOISE3.^15^ Sequences that have been denoised by these packages are referred to by different names: amplicon sequence variants,^13^ sub-OTUs^14^ or zero-radius OTUs.^15^ In this study, these synonymous terms are called ‘sequence variants’ (SVs). For taxonomy assignment of SVs, reference databases for rRNA, such as SILVA for the 16S and 18S rRNA genes,^16^ RDP for the 16S rRNA and fungal 28S rRNA genes,^17^ and PR2 for the 18S rRNA gene,^18^ are well maintained and often used for microbiome studies.

The per-sample sequence depth that is required for amplicon sequencing differs between samples but typically ranges from 10 to 100 K reads.^19^ On the other hand, high-throughput DNA sequencers produce much greater numbers of reads (a maximum of 25 M reads for MiSeq and 5,000 M reads for HiSeq4000). Therefore, researchers use index adaptors to multiplex the different DNA libraries and sequence the pooled libraries in a single run,^20^ which greatly reduces sequencing cost per sample. The dual index of a DNA library is the fundamental technique for Illumina sequencers, which are the most commonly used platforms for amplicon sequencing.^21^ The DNA libraries for Illumina sequencers contain P5 and P7 adapters at DNA ends, and these adapters are required for cluster generation for bridge PCR in the flowcell (Supplementary Fig. S1A). Dual index sequences of 8 or 10 bp for multiplexing are inserted next to the P5 and P7 adaptors (Supplementary Fig. S1A). After cluster generation, sequencing primers hybridize at their specific positions within the adaptor, followed by attachment of a dye terminator and capture of fluorescence images; each end and two index regions of the DNA libraries are sequentially sequenced (Supplementary Fig. S1A). The sequence reads produced are demultiplexed based on the combination of dual index sequences. Various dual index systems are commercially available or have been developed by researchers,^22^^,^^23^ which often utilize the same adapter sequences of the Nextera or TruSeq kit from Illumina (Supplementary Fig. S1A).

Many studies have been conducted for the validation and comparison of the steps of amplicon sequencing,^10^^,^^24–27^ which include the sequencing platform, target regions, sample preparation techniques and software packages and their parameters. However, the validation of combinations of dual index adaptors is often overlooked and rarely performed by sequencing on the Illumina platform. In this study, we report that a specific combination of dual index adaptors decreases the quality of sequences generated with the MiSeq platform. The decrease in quality was indeed found to affect all reads and positions (in either orientation) of the sequence reads containing a specific index combination, which was probably a consequence of impaired cluster generation and/or subsequent sequencing. We estimated the effect of the quality decrease during a community analysis of the 16S and 18S rRNA amplicons with the use of the dada2 plugin in QIIME2,^28^ which is one of the most common open software packages for sequence analysis. The diminished sequence quality was found to result in a significant decrease in α-diversity and could not be recovered by tuning the denoising parameters. Our data underscore the importance of quality validation of sequence reads when developing dual index technologies for Illumina platforms and suggest cautious interpretation of microbiome data containing low-quality sequence reads.

## 2. Materials and methods

### 2.1. Library preparation of *Escherichia coli* DNA

Genomic DNA of *Escherichia coli* strain HST08 (Takara Bio Inc.), which was originally derived from *E. coli* strain K-12, was used for the preparation of a sequence library containing diverse insert sequences. *Escherichia coli* cells were grown in Luria–Bertani medium and harvested by centrifugation. Cells were lysed in a buffer (10 mM Tris-HCl, pH 8.0, 1 mM EDTA) containing 1% (w/v) SDS, extracted with Tris-buffered phenol and centrifuged at 8,000 × *g* at 20°C for 5 min for phase separation. DNA was isolated from the upper/aqueous phase using the DNeasy blood and tissue kit. The concentration of the purified DNA was 19.0 ng/μl, as quantified with the Qubit dsDNA BR Assay kit (Thermo Fisher Scientific). Tagmentation of *E. coli* genomic DNA was performed using the Nextera XT DNA sample prep kit (Illumina) with the following modifications: the amount of input DNA was reduced from 1 to 0.1 ng, the tagmentation reaction time was increased from 5 to 15 min and 2× tagmentation buffer from the kit was replaced with 5× tagmentation buffer of the Nextera Mate Pair Library Preparation kit (Illumina). Tagmentation was stopped with addition of NTB and NPM buffers (Nextera XT DNA sample prep kit), and the libraries were indexed with 19 combinations of the index adaptor containing either N704 or S507 using the Nextera XT index kit set A v2 (Illumina) with 15 cycles of index PCR using KOD FX Neo DNA polymerase (Toyobo). Thermal cycling for index PCR was as follows: initial denaturation at 94°C for 2 min, then 30 cycles of denaturation at 98°C for 10 s, annealing at 55°C for 30 s and extension at 68°C for 30 s, with a final extension step at 68°C for 5 min. DNA in each amplified library was purified with 0.55 volumes of AMPure XP beads. The size distribution of each library was checked with the High Sensitivity DNA kit (Agilent) on a Bioanalyzer 2100 (Agilent).

### 2.2. Library preparation of the 16S and 18S rRNA amplicons

As the sequencing target for the 16S and 18S amplicons, we chose an algal mat (sample S1) that we previously collected from a freshwater lake in Antarctica.^29^ The DNA of S1 was purified previously^29^ (10.7 ng/μl, as quantified with the Qubit dsDNA BR Assay kit). The V3–V4 region of 16S rRNA and V7–V8 region of 18S rRNA were amplified with the primer pair 341F and 805R^30^ and F1183 and R1631,^11^ respectively (we previously reported the full sequences of these primers^29^). Enzyme and PCR conditions were the same as those for index PCR of *E. coli* DNA except that there were 30 PCR cycles. The first amplicon PCR products were purified with 0.8 volume of AMPure XP beads and eluted with 10 mM Tris-HCl (pH 8.5). The second index PCR was performed with eight cycles using the Nextera XT index kit v2 set A (Illumina) with the aforementioned PCR conditions. Amplified libraries were purified by addition of 1.12 volumes of AMPure XP beads and eluted with 10 mM Tris-HCl (pH 8.5). The DNA in each library was quantified using the Qubit dsDNA HS Assay kit (Thermo Fisher Scientific). The size distribution of libraries was investigated using the Agilent High Sensitivity DNA kit on the Bioanalyzer 2100. The two libraries of 16S rRNA amplicons indexed with N504/S507 and N503/S507 combinations were subjected to direct sequencing on 3730xl DNA analyzer (Applied Biosystems) by Macrogen Japan using primer P5 (5′-ATACGGCGACCACCGAGATC) or P7 (5′-CAAGCAGAAGACGGCATACGAG) (Supplementary Fig. S1C). The sequence libraries for the *E. coli* genome and the 16S rRNA and 18S rRNA amplicons were pooled and sequenced for 300 bp of both ends using the MiSeq reagent kit v3 (600 cycles; Illumina) with the MiSeq instrument (Illumina). Data were processed using Real-Time Analysis ver. 1.18.54 and MiSeq Control Software ver. 2.6.21. Cluster density was 742 K/mm^2^, the cluster passing filter was 90.3% and the aligned PhiX spike-in was 12.3%. The produced sequence reads were demultiplexed based on the combination of dual index adapters with the MiSeq Control Software.

### 2.3. Data analysis

To obtain the sequence reads derived from *E. coli* DNA, demultiplexed sequences were mapped to the reference genome sequence of *E. coli* K-12 substrain W3110 in the RefSeq database (GCF_000010245.2_ASM1024v1_genomic.fna) using bowtie2 with default parameters.^31–33^ Uniquely mapped reads were obtained using samtools view command with the -q 4 option in samtools ver. 1.10,^34^ which also removed the 16S and 18S rRNA amplicons that could be mapped multiple times to four copies of the rRNA operons in *E. coli*. Ranges of 125,389–270,435 read 1 sequences and 118,612–266,374 read 2 sequences were obtained as sequence reads uniquely mapped to the *E. coli* genome (Supplementary Table S2). Reference sequences were corrected with pilon ver. 1.23 with—fix bases option.^35^ Paired reads were extracted from uniquely mapped reads using fastq_pair,^36^ and read length was trimmed to 300 bp using seqtk ver. 1.3 (https://github.com/lh3/seqtk). The processed paired reads were mapped to the corrected *E. coli* genome using bowtie2, and sequence error rate was calculated using the samtools stats command. Per-read and per-base Phred quality scores were calculated using FastQC v0.11.9.^37^ The distribution of the mean expected errors per sample was calculated using the command vsearch –fastq_eestats2.^38^ The results files exported from these programmes were processed using custom scripts in Perl ver. 5.24.4 or R ver.3.6.1.

To obtain the sequence reads for the 16S or 18S rRNA amplicon, the 5′ sequence of each read was searched in the demultiplexed sequences. We used cutadapt v2.9^39^ with the options -g ^CCTACGGGNGGCWGCAG and -G ^GACTACHVGGGTATCTAATCC for the 16S rRNA amplicon and options -g ^AATTTGACTCAACACGGG and -G ^TACAAAGGGCAGGGACG for the 18S rRNA amplicon. Additionally, we utilized discard-untrimmed and no-indels options for both amplicons. These options extract sequence reads that harbor 5′-anchored amplicon primers in both reads, remove the primer sequence and export the sequence reads of uniform length: 284 bp of read 1 and 280 bp of read 2 for the 16S rRNA amplicon, and 283 bp of read 1 and 284 bp of read 2 for the 18S rRNA amplicon. The total number of read pairs ranged from 35,311 to 82,408 for the 16S rRNA amplicon and from 44,785 to 101,078 for the 18S rRNA amplicon (Supplementary Table S2). The sequence reads of the 16S and 18S rRNA amplicons were imported to the environment of QIIME2 ver. 2020.2.^40^ Forward and reverse reads were joined, denoised and checked for chimeras using the dada2 plugin with quality filtering thresholds of [6, 8] for the [–p-max-ee-f, –p-max-ee-r] parameters and overlap length thresholds of [260, 210] for the [–p-trunc-len-f, –p-trunc-len-r] parameters for both the 16S and the 18S rRNA analyses. For the optimization of these parameters, we compared the values of [2, 4], [4, 8], [6, 8] and [8, 10] for the [–p-max-ee-f, –p-max-ee-r] parameters and values of [250, 200], [260, 210] and [270, 220] for the [–p-trunc-len-f, –p-trunc-len-r] parameters. This optimization was performed with dada2 ver. 1.14 on R to investigate the number of denoised forward and reverse reads because the dada2-plugin (ver. 1.10) in QIIME2 reports the number of denoised reads only for forward reads in dada2-stats file.

For denoising using deblur plugin, the adapter-removed reads were trimmed at their 3′ end to yield read 1 with 260 bp and read 2 with 210 bp using seqtk. Paired reads were joined using the vsearch join-pairs command and denoised using the deblur denoise-16S or denoise-other (for 18S) command with –p-trim-length 400 –p-min-reads 2 options. Reference sequences of 18S rRNA clustered at 90% identity in SIVA ver.132 (silva_132_90_18S.fna) were used for the positive filtering of the 18S rRNA analysis. Rate of the number of unique reads in total merged reads was analysed using the command vsearch-derep_fulllength. For OTU clustering, joined reads were processed with the vsearch dereplicate-sequences and cluster-features-*de-novo* commands at thresholds of 0.97 for 16S rRNA and 0.99 for 18S rRNA, which are followed by *de novo* chimera removal with the vsearch uchime-denovo command. Low-abundance OTUs were then removed with the feature-table filter-features command in QIIME2 with –p-min-frequency 2 or 10 option.

Taxonomy of the SVs was assigned using a feature-classifier plugin that was trained with taxonomy information of majority_taxonomy_7_levels.txt with 99% clustering in the SILVA database ver. 132 (https://www.arb-silva.de/download/archive/)^16^ for the 16S rRNA analysis or pr2_version_4.12.0_18S_mothur.tax of the Protist Ribosomal Reference database (PR2) (https://github.com/pr2database/pr2database/releases)^18^ for the 18S rRNA analysis. Rarefaction curves were plotted using the rarecurve function in the vegan package ver. 2.5.6. For α-diversity analyses, non-chimeric SVs were rarefied to an even depth of 16,958 and 28,018 sequences for the 16S and 18S rRNA genes, respectively, using the rarefy_even_depth function of the phyloseq package ver. 1.28.0 in R.^41^ Shannon and Simpson diversity indices^42^ were calculated with the estimate_richness function of the phyloseq package. The Grubbs test^43^ was performed using the outliers package ver. 0.14 in R.^44^ Plots were visualized using the phyloseq or ggplot2 ver. 3.2.1 packages in R.^41^^,^^45^ All sequence data for *E. coli* and the 16S and 18S rRNA amplicons were deposited in DDBJ with accession number DRA010058.

## 3. Results and discussion

### 3.1. A specific combination of indexes decreases the quality of the outputted sequence data

**Figure 1:**
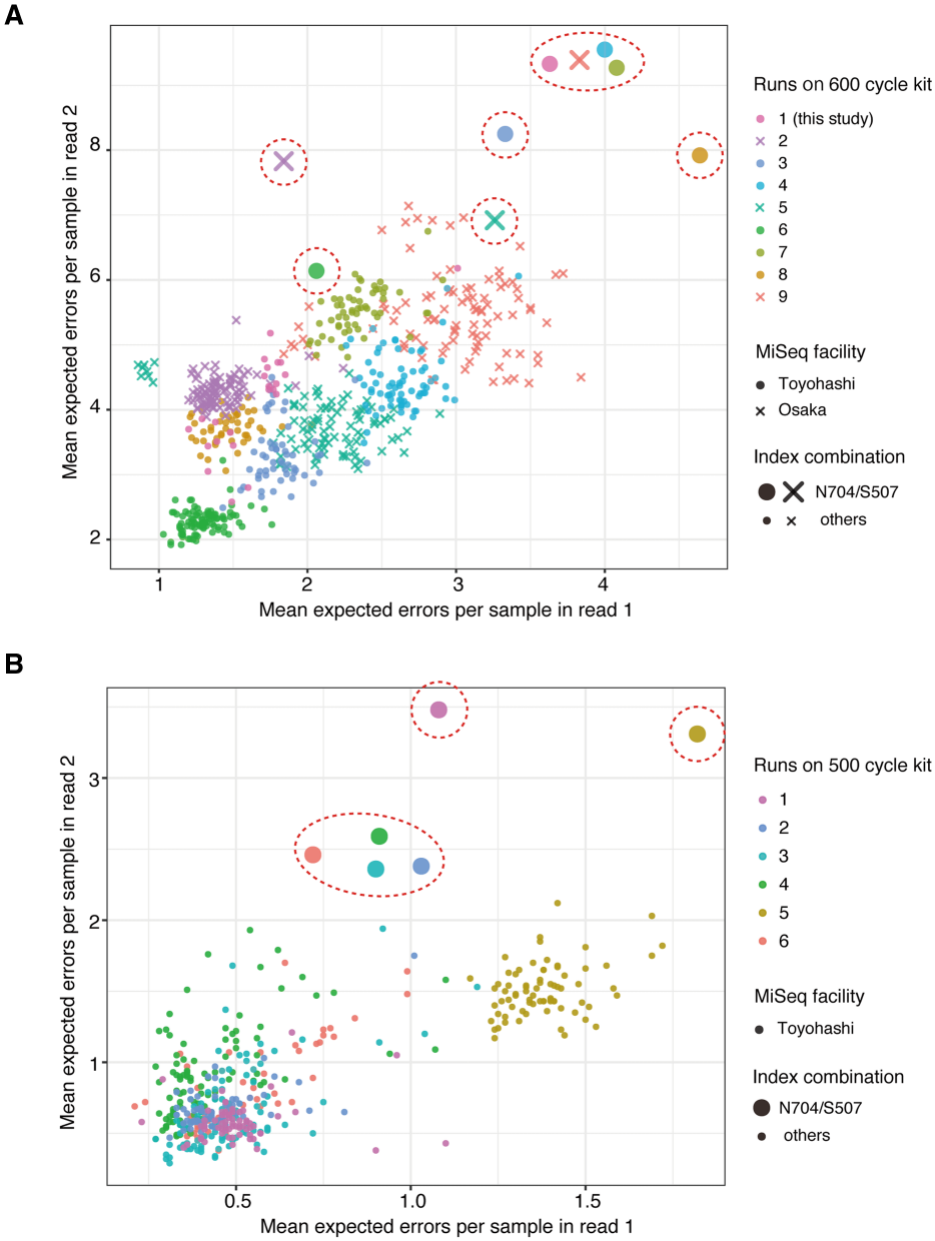
Effects of the combination of dual index adaptors on sequence quality with MiSeq. Distribution of the arithmetic mean of expected error per sample is shown for read 1 and read 2 of paired-end sequences. Runs were performed with the different MiSeq sequencers operated at Toyohashi University of Technology (Toyohashi) or Osaka Prefecture University (Osaka) with the MiSeq reagent kit for 600 cycles (A) or 500 cycles (B). Samples in different runs are colored accordingly. Samples with the N704 and S507 index combination are enclosed with a red dashed line.

For the samples we subjected to amplicon sequencing on MiSeq, we frequently observed that one of the samples had consistently lower sequence quality compared with the other samples. Figure 1 shows the typical data for per-sample sequence quality for nine runs with the 600 cycle kit, producing 300 bp × 2 paired reads (Fig. 1A), and six runs with the 500-cycle kit, producing 250 bp × 2 paired reads (Fig. 1B).^46^ In Illumina paired-end sequencing, read 1 has higher quality than read 2. Notably, decreases of sequence quality were observed in both read 1 and read 2 of the aforementioned low-quality sample in each run (Fig. 1A and B, enclosed circles). Runs with the 500-cycle kit produced higher sequence quality than runs with the 600-cycle kit, as sequence quality gradually decreased towards the end of the sequence read. The odd decrease in sequence quality was observed in runs with both types of kits (Fig. 1A and B). The quality decrease was observed in the two different MiSeq instruments operated in the two different facilities: Toyohashi University of Technology and Osaka Prefecture University (Fig. 1A). Samples in these runs were derived from different environments such as seawater, rat feces, human skin and microbial mats, and thus different samples contained different regions such as region V4 and the V3–V4 and V1-V2 regions of 16S rRNA, the V7–V8 region of 18S rRNA, the gene *nifH* and combinations of these loci (Supplementary Table S1). These points suggest that the observed decrease in sequence quality was not caused by the type of sequence kit, instrument-specific issue or sample origin. We performed extensive trouble shooting of this phenomenon and ultimately surmised that samples with low-sequence quality commonly contained a specific combination of dual index adaptors, namely N704 and S507 indexes, in the Nextera index kits (Illumina) (Fig. 1A and B). As Illumina is not responsible for providing support for amplicon sequencing, we carried out a detailed investigation of the effects of the N704/S507 index combination.

**Figure 2:**
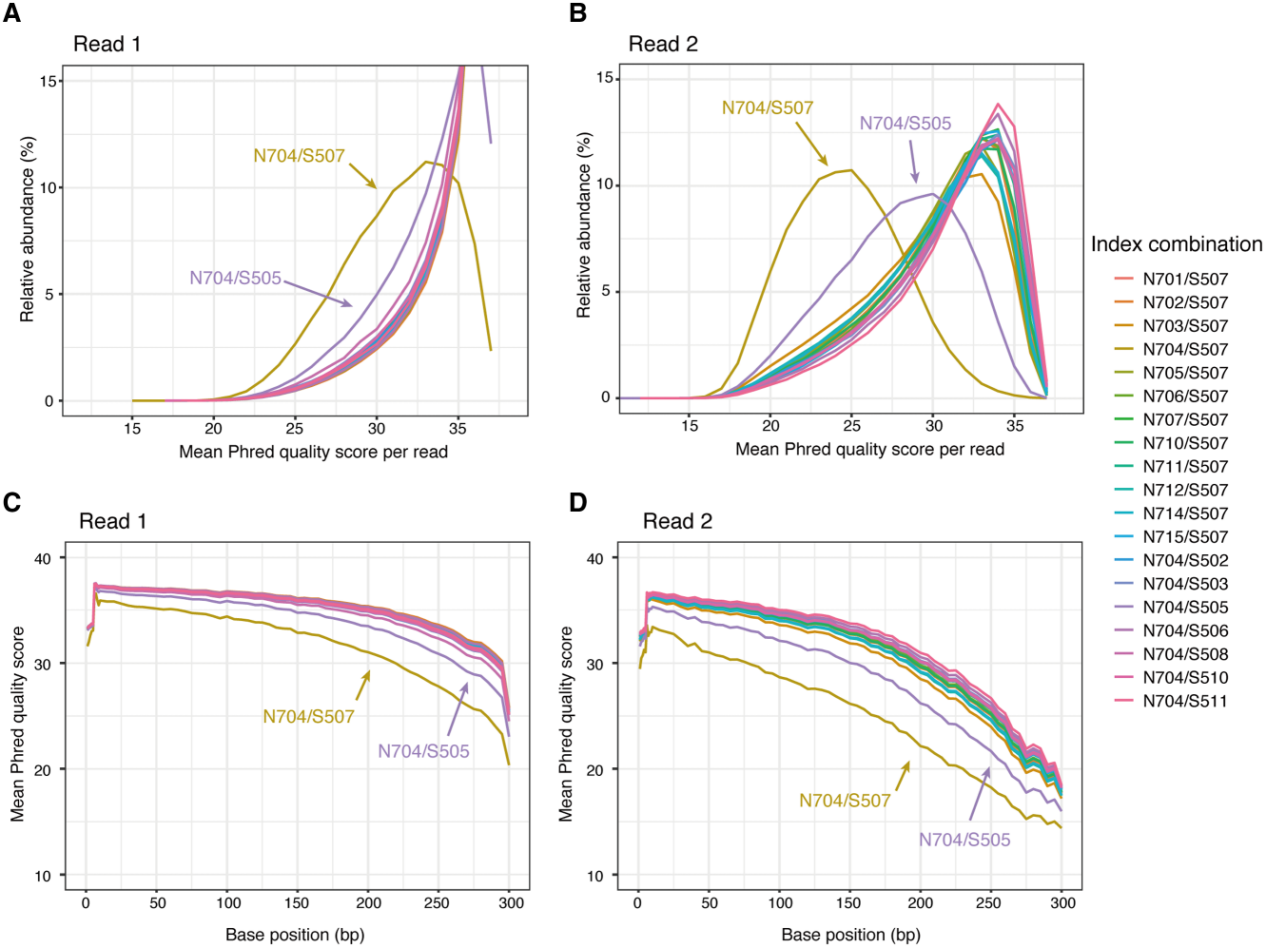
Effects of the combination of dual index adaptors on sequencing of *E. coli* genomic DNA. The distribution of per-read Phred quality scores is shown for read 1 (A) and read 2 (B) of sequence reads for *E. coli* genomic DNA that were fragmented by Nextera transposase. Distribution of per-base Phred quality score positions is shown for read 1 (C) and read 2 (D). Sequence qualities for 19 samples containing either the N704 or the S507 adaptor are colored accordingly.

To investigate the effect of the N704/S507 index combination on sequence quality, we sequenced genomic DNA of *E. coli* that had been randomly fragmented using Nextera transposase and indexed with a total of 19 index combinations containing either N704 or S507 (Supplementary Fig. S2 and see Section 2 for experimental design). The distribution of per-read Phred quality (*Q*) scores revealed that decreased sequence quality was observed in both read 1 and read 2 with the N704/S507 combination but not with the other index combinations (Fig. 2A and B). The N704/S507 combination showed single peaks at Q33 for read 1 and at Q25 for read 2, whereas most of the other combinations showed single peaks at Q35 for read 1 and at Q33–34 for read 2 (Fig. 2A and B). Per-base Phred quality scores revealed that the decrease of the sequence quality for N704/S507 occurred at all positions of read 1 and read 2 (Fig. 2C and D). These results indicate that (i) the decrease of sequence quality was not caused by the sequence inserted in the amplicons but rather by the specific combination of N704/S507 dual index adaptors, (ii) the decrease of sequence quality was global in the produced reads and (iii) the decrease did not occur when either the N704 or the S507 adaptor was used in combination with any other index adaptor. We investigated the rate of mismatches of the sequence reads mapped to the *E. coli* genome and confirmed that the sequence error rate indeed increased by ∼2-fold in the N704/S507 combination (Supplementary Fig. S3). These results revealed that the specific combination of N704 and S507 adaptors caused a decrease of sequence quality and an increase of actual sequence errors for MiSeq-outputted sequences. Notably, the N704/S505 combination led to slightly lower per-read and per-base Phred quality scores compared with the other samples (Fig. 2A–D and Supplementary Fig. S3A), reinforcing the idea that this combination was responsible for the observed decrease in sequence quality.

### 3.2. Effects of index combination on sequencing and denoising of the 16S and 18S rRNA amplicons

**Figure 3:**
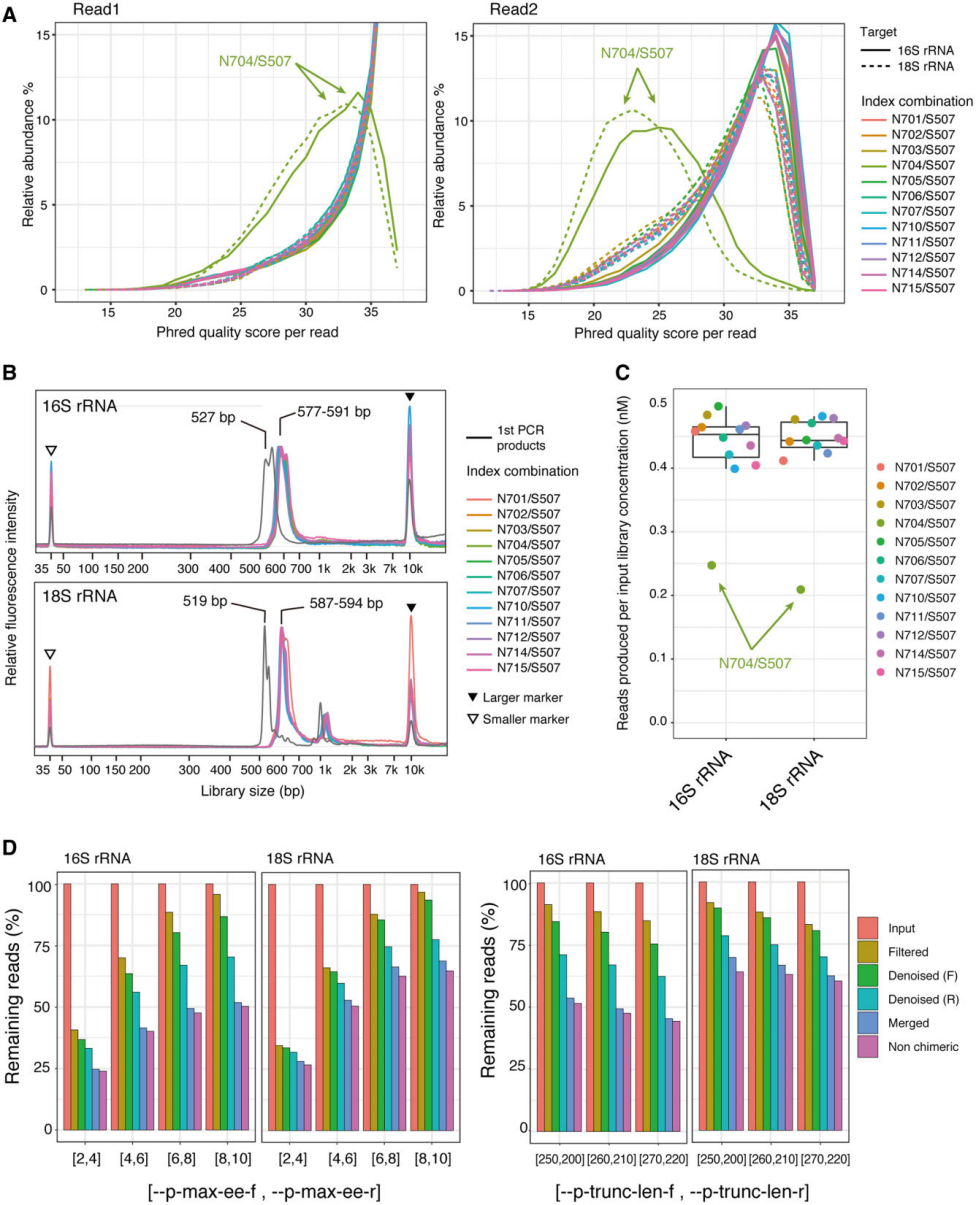
Effects of the combination of dual index adaptors on the analysis of the 16S and 18S rRNA amplicons. (A) The distribution of per-read Phred quality scores is shown for read 1 (left) and read 2 (right) of sequence reads for 16S (solid line) and 18S rRNA amplicons (dashed line). (B) Distribution of fragment sizes for the 16S rRNA (upper) and 18S rRNA amplicons (lower) from the Bioanalyzer 2100. The larger marker (filled triangle) and smaller marker (open triangle) are indicated. (C) Sequence yield for each library was estimated based on the relationship between the total reads and the concentration of the input library. For (A–C), the plots for the total of 12 samples with different index combinations are colored accordingly. (D) Optimization of parameters used for the denoising of the plugin dada2 for filtering quality (two plots, left) and trimming length (two plots, right) for the 16S and 18S rRNA amplicons. The abundance of the reads remaining after each process was scaled to the number of input sequence reads. Input, filtered, denoised forward (F), denoised reverse (R), merged and non-chimeric reads are colored accordingly.

We next investigated the effect of the index combination on amplicon sequencing of the V3–V4 region of the 16S rRNA gene and the V7–V8 region of the 18S rRNA gene. Our protocol for the 16S rRNA V3–V4 region corresponded to the protocol provided by Illumina.^47^ We utilized an environmental sample from an algal mat collected from a freshwater lake in Antarctica, whose community structure was revealed in our recent study using the primer sets mentioned above.^29^ To investigate the effects of index combination, aliquots of first-amplicon PCR products derived from the algal mat DNA were subjected to a second index PCR with 12 index combinations of the N7xx series and S507 (Supplementary Fig. S2). As observed for the sequencing of *E. coli* genomic DNA, the decrease of per-read Phred quality score was observed in both read 1 and read 2 for both the 16S and 18S rRNA amplicons (Fig. 3A). Fragment analysis showed that the library size increased by 50–75 bp after index PCR for all samples, and no primer dimers were detected (Fig. 3B). We performed Sanger sequencing of the 16S rRNA amplicon libraries indexed with N504/S507 or N503/S507 (control) combinations using custom primers that anneal to the P5 or P7 region and confirmed chromatogram for the adaptor sequences (Supplementary Fig. S1C). These data indicated that the decrease of sequence quality in the library with the N704/S507 combination was not caused by contamination of primer dimers or by heterogeneity of the DNA library owing to inefficient index PCR but rather by sequencing processes inherent in MiSeq. The relationship between the number of reads and the concentration of the input library showed that sequence production yields decreased in libraries with the N704/S507 combination for both the 16S and the 18S rRNA amplicons (Fig. 3C). This implies that the decrease of sequence quality is caused by a partial deficiency in cluster generation and/or subsequent sequencing processes in the flowcell.

We attempted to mitigate the decrease in both the number and the quality of sequence reads with the N704/S507 combination by tuning parameters in the denoising process of the dada2 plugin in QIIME2.^28^ In the current version of QIIME2, denoising using the dada2 plugin is performed with the following steps^13^: (i) the sequence reads of read 1 and read 2 are filtered based on the quality (maximum number of expected errors) and trimmed at the 3′ end of each read at a specified length; (ii) sequence errors in read 1 and read 2 are removed separately based on the quality, hamming distance and abundance of the reads, and singleton reads are also removed during this process; (iii) read 1 and read 2 pairs are merged, having at least a 12-bp overlap, and read pairs containing any mismatches are discarded and (iv) *de novo* chimera detection is performed, and chimeric read pairs are discarded. We investigated the number of reads remaining after each process using four different values for the maximum expected error, namely, [2, 4], [4, 6], [6, 8] and [8, 10] for [–p-max-ee-f, –p-max-ee-r]. These values were determined based on the distribution of expected errors in read 1 and read 2 (Supplementary Fig. S4). In the samples with the N704/S507 combination, increasing the values of [–p-max-ee-f, –p-max-ee-r] improved the relative abundance of filtered reads, but substantial numbers of reads were removed during denoising and merging processes, and non-chimeric reads essentially plateaued at ∼50% of input reads (Fig. 3D). On the other hand, samples with other index combinations remained at ∼70–80% of input reads as non-chimeric reads for any values of [–p-max-ee-f, –p-max-ee-r] parameters (Supplementary Fig. S5). These data suggested that denoising did not fully remove sequence errors and that the resultant singleton reads and/or mismatches in the paired reads were responsible for the decrease in the number of non-chimeric reads. The length of overlap of the paired reads for 460-bp amplicons was estimated at ∼50 bp when the values of [260, 210] were used for [–p-trunc-len-f, –p-trunc-len-r]. A change of these parameters to [250, 200] (∼30-bp overlap) or [270, 220] (∼70-bp overlap) did not substantially change the abundance of the non-chimeric reads with all examined index combinations (Fig. 3D and Supplementary Fig. S6). This suggested that the reduction of reads was caused mainly by the denoising process rather than by mismatches resulting from the merging process. Notably, the reduction of reads occurred in the denoising of the reverse reads rather than the forward reads, underscoring the inherent difficulty of denoising low-quality sequences. Data sets with the parameter [6, 8] for [–p-trunc-len-f, and –p-trunc-len-r] and [260, 210] for [–p-trunc-len-f, –p-trunc-len-r] were used for further analysis of community structures of the 16S and 18S rRNA amplicons. We also investigated the rate of the number of unique reads in the total merged reads without denoising to estimate the actual sequence error rate. The rate of the unique reads in the N704/S507 combination was 93% in both 16S and 18S rRNA amplicons, which was substantially higher than 58% for 16S rRNA and 50% for 18S rRNA in the average of rest combinations and reflects the increase of the actual sequence error rate (Supplementary Fig. S3B).

### 3.3. Effect of index combination on community structure of the 16S and 18S rRNA amplicons

**Figure 4:**
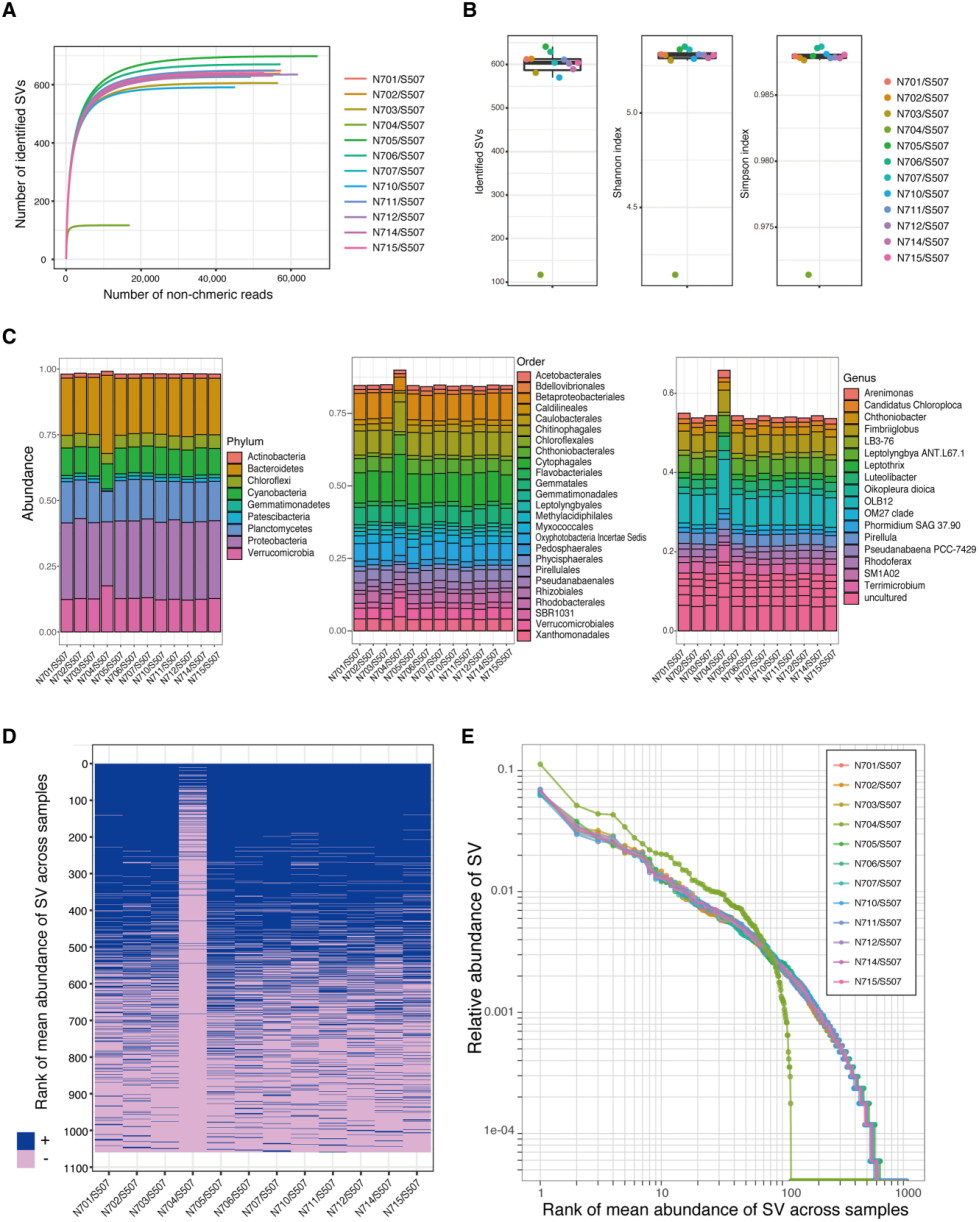
Effects of the combination of dual index adaptors on the community analysis of the 16S rRNA amplicon denoised using dada2. (A) Rarefaction curves for the number of identified SVs obtained using dada2 plugin for different numbers of non-chimeric reads. (B) The α-diversity of the identified SVs (left), Shannon index (centre) and Simpson index (right). (C) Community structures of the 16S rRNA amplicons are shown at the phylum (left), order (centre) and genus (right) levels with reference to the SILVA ver. 132 database. SVs were agglomerated to each level, and taxa below 1% are not shown. The names of taxa are colored accordingly. (D) The presence (blue) or absence (pink) of each SV is shown in a heatmap, in which SVs are sorted in decreasing order of mean abundance across 12 samples. (E) Relationship between relative abundance and rank of the SVs. Plots of the 12 samples with different index combinations are colored accordingly for (A), (B) and (E).

Rarefaction curves for the number of identified SVs of 16S rRNA amplicon using dada2 plugin revealed that samples with the N704/S507 combination reached a plateau at a substantially lower number of SVs compared with the other index combinations (Fig. 4A). The decrease of α-diversity for the N704/S507 combination was significant in the Grubbs test for the identified SVs (*P *=* *3.121e − 9), Shannon index (*P *=* *1.521e − 12) and Simpson index (*P *=* *1.675e − 12) for reads rarefied to 16,958 non-chimeric reads (Fig. 4B). The phylum-level composition of the 16S rRNA amplicons based on the SILVA ver. 132 database showed an increase of Bacteroidetes and Verrucomicrobia and a decrease in Planctomycetes and Proteobacteria compared with the samples having other index combinations (Fig. 4C, left). The major taxa (>1%) at the order and genus levels of classification were detected in all 12 samples, but their relative abundances were increased when the N704/S507 combination was used, as compared with the other index combinations (Fig. 4C, centre and right). Heatmap analysis of the presence or absence of SVs, which were sorted in decreasing order of mean abundance across all samples, revealed that most of the low-abundance SVs and even some of the abundant SVs could not be detected in samples with the N704/S507 combination (Fig. 4D). The plot of relative abundance of each SV and its rank demonstrated the increase of high-rank SVs and decrease of low-rank SVs in samples with the N704/S507 combination (Fig. 4E). Thus, the use of the N704/S507 combination substantially decreases the sensitivity of detection of the minor SVs and alters the overall composition and α-diversity of the samples in amplicon sequencing.

**Figure 5:**
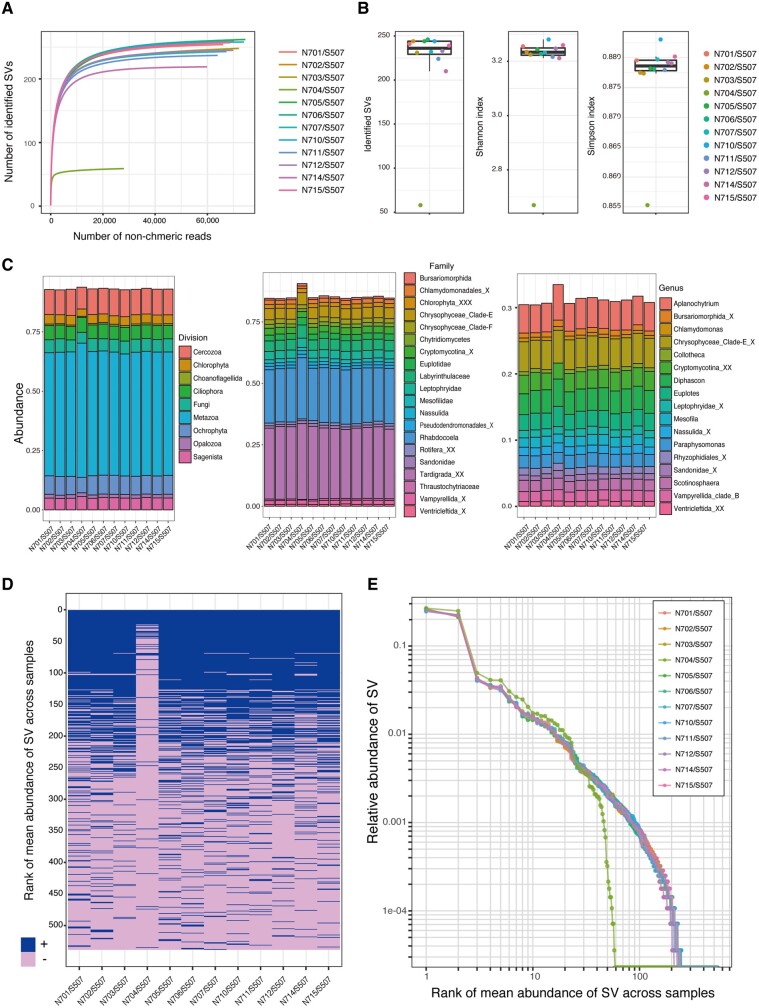
Effects of the combination of dual index adaptors on the community structure of the 18S rRNA amplicon denoised using dada2. (A) Rarefaction curves for the number of identified SVs obtained using dada2 plugin for different numbers of non-chimeric reads. (B) The α-diversity of the identified SVs (left), Shannon index (centre) and Simpson index (right). (C) Community structures of the 18S rRNA amplicons are shown at the division (left), family (centre) and genus (right) levels with reference to the PR2 database, in which taxa below 0.5% are not shown. Taxa are colored accordingly. (E) The presence (blue) or absence (pink) of each SV is shown in a heatmap, in which the SVs are sorted in decreasing order of mean abundance across 12 samples. (E) Relationship between relative abundance and rank of the SVs. Plots of the 12 samples with different index combinations are colored accordingly for (A), (B) and (E).

We also investigated the effect of index combination in the analysis of 18S rRNA amplicons, which are less diverse than the 16S rRNA amplicons. Rarefaction analysis with dada2 plugin revealed that samples with the N704/S507 combination reached a plateau at a lower number of SVs compared with the other index combinations, as was the case for the16S rRNA amplicons (Fig. 5A). The decrease of α-diversity for the N704/S507 combination was significant in the Grubbs test for the identified SVs (*P *=* *1.477e − 7), Shannon index (*P *=* *7.578e − 10) and Simpson index (*P *=* *3.847e − 7) at a read depth rarefied to 28,018 (Fig. 5B). Division-level composition of the 18S rRNA amplicons based on the PR2 database revealed an increase of Metazoa and a decrease of Cercozoa compared with the samples of the other index combinations (Fig. 5C, left). The major taxa (>0.5%) at the family and genus levels of classification were detected in all 12 samples, but their relative abundances were increased in the N704/S507 combination compared with the other index combinations (Fig. 5C, centre and right). Heatmap analysis revealed that low-abundance SVs could not be detected in samples with the N704/S507 combination and that this failure of detection was responsible for the observed increase in the relative abundance of high-rank SVs in those samples (Fig. 5D and E). Thus, the use of the N704/S507 combination substantially decreases the sensitivity of minor SVs in amplicon sequencing for both the 16S and the 18S rRNA genes.

**Figure 6:**
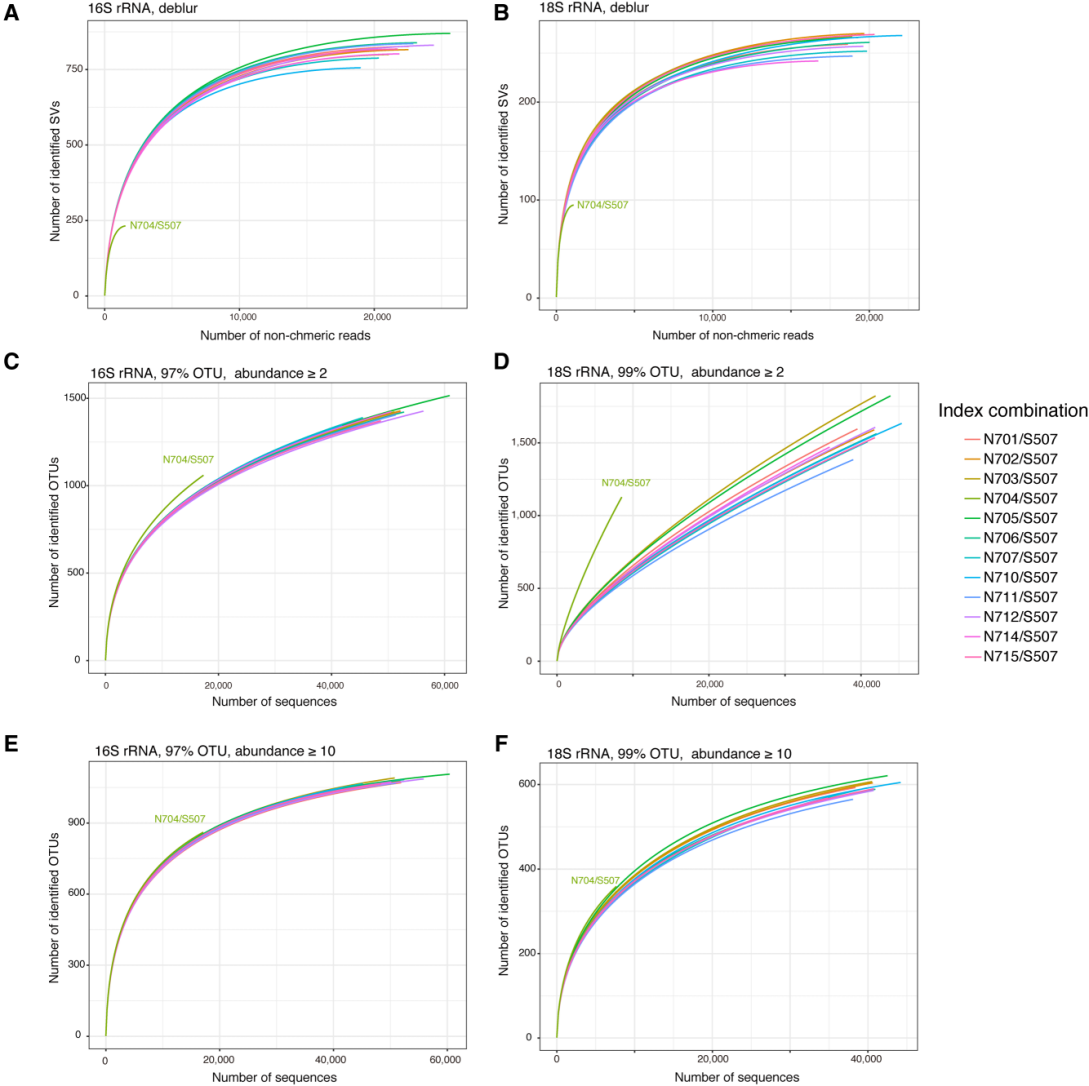
Rarefaction curve analyses for SVs denoised using deblur and clustered OTUs. Rarefaction curve analyses were performed for SVs denoised using deblur plugin in the 16S (A) and 18S (B) rRNA amplicons. The same analyses were performed for OTUs clustered at 97 and 99% for the 16S (C and E) and 18S (D and F) rRNA amplicons with abundance-based filtering of 2 (C and D) or 10 (E and F). Lines of the 12 samples with different index combinations are colored accordingly.

We assessed the effect of the index combination using another denoising plugin, deblur,^14^ in QIIME2. Rarefaction curve analysis of SVs obtained using deblur showed a similar result using dada2 but with a more substantial decrease in the total number of non-chimeric reads of the N704/S507 combination in the 16S and 18S rRNA amplicons (Fig. 6A and B). The differences may be due to the different denoising approaches between deblur and dada2: deblur denoises sequence errors and removes singletons per sample with a positive filtering against reference database, whereas dada2 performs denoising and singleton removal across samples without the positive filtering.^13^^,^^14^ We also performed conventional OTU clustering with typical thresholds of 97% and 99% identities for 16S and 18S rRNA amplicons, respectively. Rarefaction curves for OTUs observed at least two times across samples showed an increase in the number of the identified OTUs in the N704/S507 combination, where the 16S rRNA analysis showed moderate increase compared with the 18S rRNA analysis (Fig. 6C and D). When the threshold of the abundance filtering was raised to 10, all index combinations showed closer rarefaction curves (Fig. 6E and F). This suggests that the conventional OTU clustering with abundance filtering was more tolerant of the sequence errors and could give a more robust estimation of α-diversity compared with the denoising approach. However, the use of OTU clustering expenses the high-sequence resolution of SVs and also inflates the total number of OTUs,^13^^,^^48^ and hence users should carefully choose the methods for dealing with sequence errors.

### 3.4. Possible mechanisms for the decrease of sequence quality

Our results demonstrate that the use of the N704/S507 combination of dual index adaptors causes the observed decrease of sequence quality and sequence yield. What mechanism underlies this effect? The index sequence has a length of only 8 bp but must be responsible for the decrease of sequence quality, as this effect was found to be independent of the sequences of the insert DNA (Fig. 2). One explanation could be that, after cluster generation, the sequencing primer hybridizes not only at the proper position for sequencing but also partially to a position near the index sequences, and the consequent mixing of the emitted fluorescence in the cluster decreases the sequence quality (Figs 2A–D and 3A). Our data suggest that this is unlikely, however, because the use of either the N704 or the S507 index adapter did not affect the sequence quality. If this mechanism was responsible for the decrease of sequence quality, the decrease should have occurred in all samples containing either the N704 or the S507 index. Overall, the results indicate that the ‘interaction’ of the two index sequences, that is, N704 and S507, is responsible for the quality decrease. Notably, each end of the DNA library with the N704/S507 combination contains the sequence motif ‘AGGAGTxTCGTxGGC’ that spans the index and Nextera adaptor regions (Supplementary Fig. S1B). This fact suggests that, during primer hybridization with Illumina sequencing, the two complementary sequence motifs in the single-stranded DNA form an intra-molecular loop structure at equilibrium (Supplementary Fig. S1B). We speculate that the formation of the loop structure inhibits the hybridization of the sequencing primers for read 1 and read 2 and caused the decrease of sequencing quality. Alternatively, the complementary sequence motif may affect the efficiency of cluster generation via bridge PCR amplification of single DNA molecules. Because the sequencing mechanisms of the Illumina platform are not fully available to the public, we trust that these possibilities will be further investigated by the Illumina development team. It should be mentioned that the ‘AGGAGTC’ motif existed in the library with the N704 and S505 combination that showed slightly decreased sequence quality (Supplementary Fig. S1B), implying that the complementary sequences can lower the sequence quality in other combinations.

### 3.5. Caution for other applications

The substantial decrease of sequence quality was found to occur with the N704/S507 index combination in the Nextera XT Index kit v2 Set A, which has a maximum of 96 multiplex (combinations with the 12 indexes of the N7xx series and 8 indexes of the S5xx series). The simple solution for preventing this decrease in quality is to avoid using the N704/S507 combination and rather use the other 95 combinations, as we did not observe any substantive decrease of quality with any combination other than N704/S507 using this kit (Figs 1 and 2A–D). The Nextera DNA unique dual Index kit (maximum 384 multiplex, Illumina) utilizes a 10-bp index and does not contain any sequences corresponding to N704 or S507. However, ‘TCGTxGGC’ of the Nextera overhang sequences is responsible for the formation of 8/15 bp of the AGGAGTxTCGTxGGC motif (Supplementary Fig. S1B). This fact suggests that the use of Nextera-based dual index adaptors could lead to the formation of complementary sequences other than this motif, leading to a decrease of sequence quality. On the other hand, TruSeq-based index kits are available, for example the QIAseq 16S/ITS Index kits (maximum 1,536 multiplex, Qiagen), NEBNext Multiplex Oligos (maximum 384 multiplex, New England Biolabs) and the KAPA Dual-Indexed Adapter kit (maximum 96 multiplex, Roche). A triple-indexing design based on TruSeq adaptors has also been proposed.^22^ These TruSeq-based index adaptors do not contain complementary sequences like those found in Nextera adaptors (Supplementary Fig. S1B).

##  

Our data demonstrate the importance of validating the index combination for developing dual index systems. For example, iNext and iTru dual index systems were recently developed and have their basis in Nextera- and TruSeq-based dual index adaptors, respectively, and these sophisticated systems can establish a total of 147,456 combinations (i.e. i7 × i5: 384 × 384).^23^ Unfortunately, these systems have been validated for 208 combinations using either adaptor (i7 × i5: 160 × 1 and 1 × 48) by quantitative PCR, and only 12 combinations have been validated by sequencing *E. coli* DNA on MiSeq.^23^ Validation of either adaptor alone was found to be insufficient because the decrease of quality can be caused by a specific combination of index sequences, for example, the N704/S507 combination. Quantitative PCR has been applied to the validation of dual index kits during their development, but this method could potentially fail to detect the decrease in sequence quality. Therefore, dual index adaptors should be validated by sequencing with the Illumina platform using a control library, for example, *E. coli* DNA or mock amplicons. It is unrealistic for users to perform a sequencing run for quality check for all these index combinations, and hence we strongly encourage manufacturers and developers of dual index kits to disclose the quality of data for all index combinations by Illumina sequencing. Alternatively, users can analyse the per-read Phred quality score for samples with different index combinations using FastQC^37^ and compare their distributions in a single graph using MultiQC.^49^ Unlike sequencing of DNA or RNA libraries, amplicon sequencing utilizes a uniform insert length, and thus the quality of all outputted sequences should be similar within the same run (Fig. 1).

## 4. Conclusion

Although the Nextera XT index kit v2 is widely used in microbiome studies,^47^^,^^50–52^ it remains unclear that how many studies have utilized the N704/S507 combination because the combination of index is rarely described in the published article or deposited sequence in the database. Therefore, it is important to validate the significance of the quality decrease in the N704/S507 combination by other researchers in other facilities over the world. Our results showed that the plugin dada2 can remove sequence errors for low-quality reads,^13^ but dada2 cannot fully recover the loss of low-abundance SVs resulting from low-quality reads (Figs 4E and 5E). The plugin deblur was also ineffective to denoise the low-quality samples with the N704/S507 combination (Fig. 6A and B). The effects of such low-quality data can be mitigated by focusing on only major SVs (e.g. SVs with mean abundance >0.5%), but this narrowed focus substantially reduces the sensitivity of amplicon sequencing (Figs 4E and 5E). Conventional OTU clustering with abundance filtering can deal with the decreased sequenced quality (Fig. 6E and F), but it expenses the high-sequence resolution of SVs and inflates the number of OTUs.^13^^,^^48^ Therefore, if low-quality data are obtained for any particular sample owing to an issue with the combination of dual index adaptors, it is better to remake the sequence library and repeat the analysis with a different index combination. Finally, we suggest that researchers should be careful in denoising the samples with any index combinations containing low-quality sequence reads and interpretation of the results in microbiome studies.

## Supplementary data

Supplementary data are available at *DNARES* online.

## Accession number

DRA010058.

## Supplementary Material

dsaa017_Supplementary_Data
