# Supplementary material for: A specific combination of dual index adaptors decreases the sensitivity of amplicon sequencing with the Illumina platform
Source: DNA Res. 2020 Aug 18;27(4):dsaa017. doi: 10.1093/dnares/dsaa017 (PMC7547650; doi:10.1093/dnares/dsaa017)
Supplement: dsaa017_Supplementary_Data [file dsaa017_supplementary_data.zip › Tables S1.pdf]

Reagent kit (number of cycles)	Run No.	MiSeq facility	Index kit	Number of index combinations	Target genes of N704/S507 combination	Number of total reads of N704/S507 combination	Comments
600	1	Toyohashi University of Technology, Japan	Nextera XT index kit v2 setA	31	341F/805R (V3-V4) of 16S rRNA<br>F1183/R1631 (V7-V8) of 18S rRNA<br>Genomic DNA of *E. coli* HST08	250,716	Data acquired in the present study
600	2	Osaka Prefecture Univesity, Japan	Nextera XT index kit v2 setA	96	341F/805R of 16S rRNA	43,250	
600	3	Toyohashi University of Technology, Japan	Nextera XT index kit v2 setA	57	341F/805R of 16S rRNA	155,043	
600	4	Toyohashi University of Technology, Japan	Nextera XT index kit v2 setA	72	341F/805R of 16S rRNA<br>F1183/R1631 of 18S rRNA	162,065	
600	5	Osaka Prefecture Univesity, Japan	Nextera XT index kit v2 setA	96	341F/805R of 16S rRNA	38,905	
600	6	Toyohashi University of Technology, Japan	Nextera XT index kit v2 setA	96	341F/805R of 16S rRNA<br>F1183/R1631 of 18S rRNA	102,630	
600	7	Toyohashi University of Technology, Japan	Nextera XT index kit	58	*nifH* gene	121,814	
600	8	Toyohashi University of Technology, Japan	Nextera XT index kit v2 setA	48	341F/805R of 16S rRNA<br>F1183/R1631 of 18S rRNA	8,550	
600	9	Osaka Prefecture Univesity, Japan	Nextera XT index kit v2 setA	96	27F/338R (V1-V2) region of 16S rRNA	148,369	
500	1	Toyohashi University of Technology, Japan	Nextera XT index kit v2 setA	61	515F/806R (V4) of 16S rRNA<br>F1183/R1631 of 18S rRNA	46,120	
500	2	Toyohashi University of Technology, Japan	Nextera XT index kit v2 setA	67	515F/806R of 16S rRNA<br>F1183/R1631 of 18S rRNA	19,746	
500	3	Toyohashi University of Technology, Japan	Nextera XT index kit v2 setA	96	341F/805R of 18S rRNA	11,486	
500	4	Toyohashi University of Technology, Japan	Nextera XT index kit v2 setA	96	Speficic primer sets of Nematode 18S rRNA	23,110	
500	5	Toyohashi University of Technology, Japan	Nextera XT index kit v2 setA	76	515F/806R of 16S rRNA	12,594	
500	6	Toyohashi University of Technology, Japan	Nextera XT index kit v2 setA	63	341F/805R of 18S rRNA	48,667	
