# Supplementary material for: A specific combination of dual index adaptors decreases the sensitivity of amplicon sequencing with the Illumina platform
Source: DNA Res. 2020 Aug 18;27(4):dsaa017. doi: 10.1093/dnares/dsaa017 (PMC7547650; doi:10.1093/dnares/dsaa017)
Supplement: dsaa017_Supplementary_Data [file dsaa017_supplementary_data.zip › Tables S2.pdf]

Index combination		*E. coli*		16S V3-V4		18S V7-V8	
i7	i5	Read 1	Read 2	Read 1	Read 2	Read 1	Read 2
N701	S507	232,874	229,863	71,446	71,446	89,147	89,147
N702	S507	254,256	250,915	70,216	70,216	94,596	94,596
N703	S507	213,547	208,390	70,211	70,211	96,156	96,156
N704	S507	125,389	118,612	35,311	35,311	44,785	44,785
N705	S507	270,435	266,374	82,408	82,408	101,078	101,078
N706	S507	207,993	205,398	69,379	69,379	92,685	92,685
N707	S507	224,888	222,551	60,125	60,125	87,923	87,923
N710	S507	230,429	227,109	54,072	54,072	98,934	98,934
N711	S507	208,035	205,826	68,032	68,032	82,814	82,814
N712	S507	176,005	172,586	75,665	75,665	91,837	91,837
N714	S507	166,235	163,224	67,449	67,449	79,206	79,206
N715	S507	220,429	218,191	63,607	63,607	90,983	90,983
N704	S502	182,098	179,374	-	-	-	-
N705	S503	164,733	162,506	-	-	-	-
N706	S505	214,945	209,392	-	-	-	-
N707	S506	293,976	291,139	-	-	-	-
N708	S508	161,747	159,559	-	-	-	-
N709	S510	155,525	153,395	-	-	-	-
N710	S511	300,481	298,368	-	-	-	-
Minimum number of reads		125,389	118,612	35,311	35,311	44,785	44,785
Maxmum number of reads		270,435	266,374	82,408	82,408	101,078	101,078
Mean number of reads		210,876	207,420	65,660	65,660	87,512	87,512
