# Supplementary figures and images for: A specific combination of dual index adaptors decreases the sensitivity of amplicon sequencing with the Illumina platform
Source: DNA Res. 2020 Aug 18;27(4):dsaa017. doi: 10.1093/dnares/dsaa017 (PMC7547650; doi:10.1093/dnares/dsaa017)

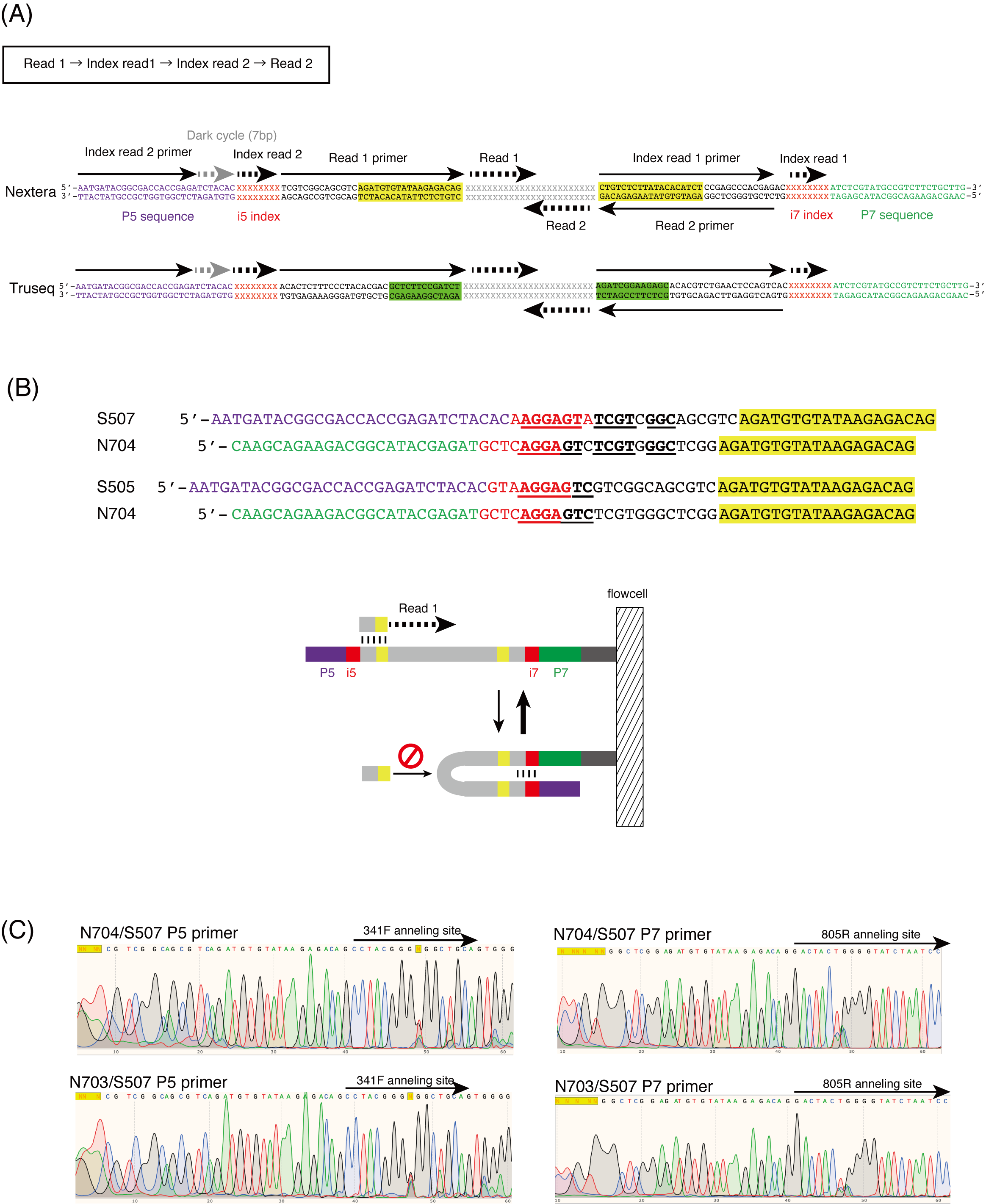

Supplement: dsaa017_Supplementary_Data [file dsaa017_supplementary_data.zip › Fig S1.tif]

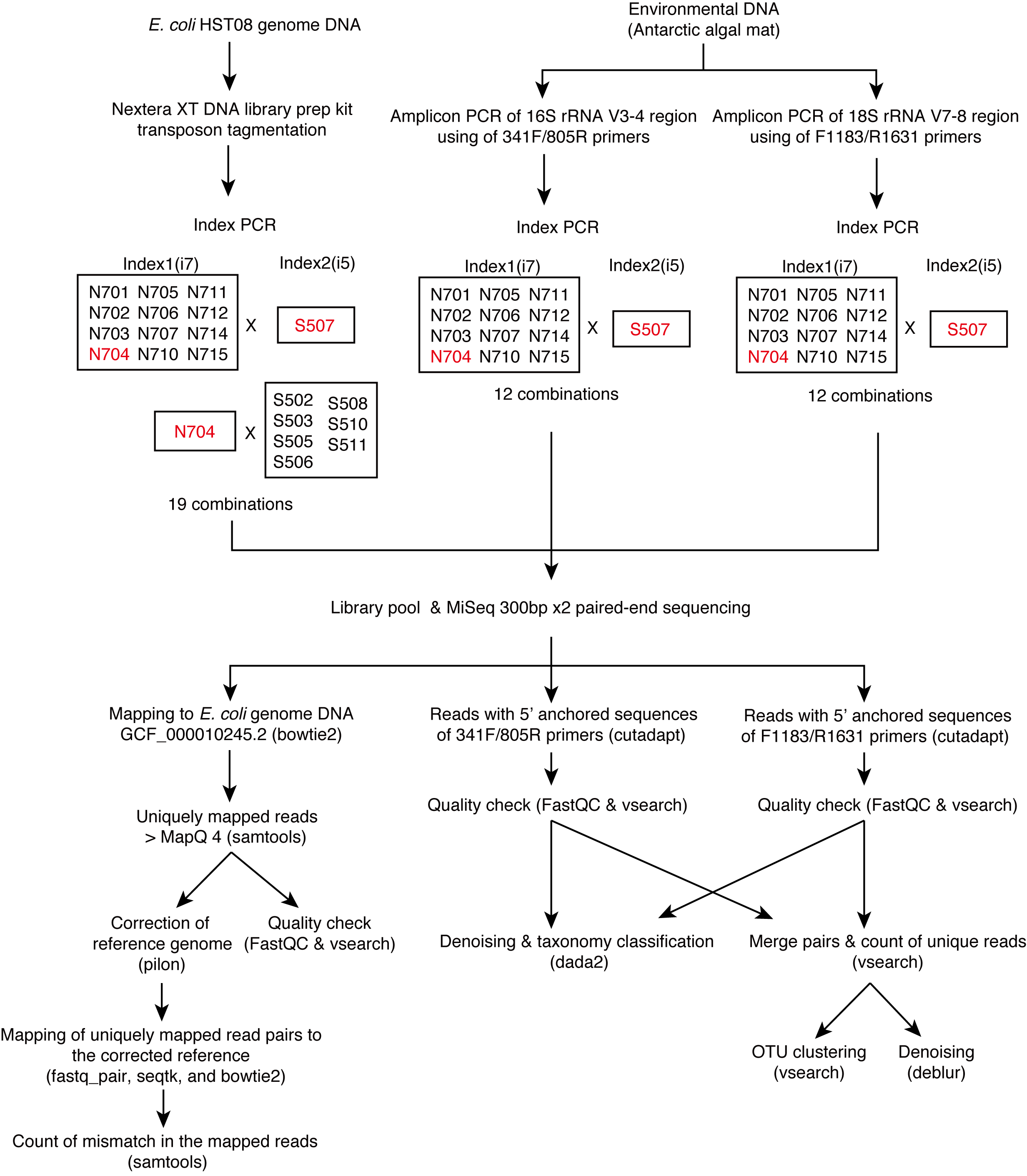

Supplement: dsaa017_Supplementary_Data [file dsaa017_supplementary_data.zip › Fig S2.tif]

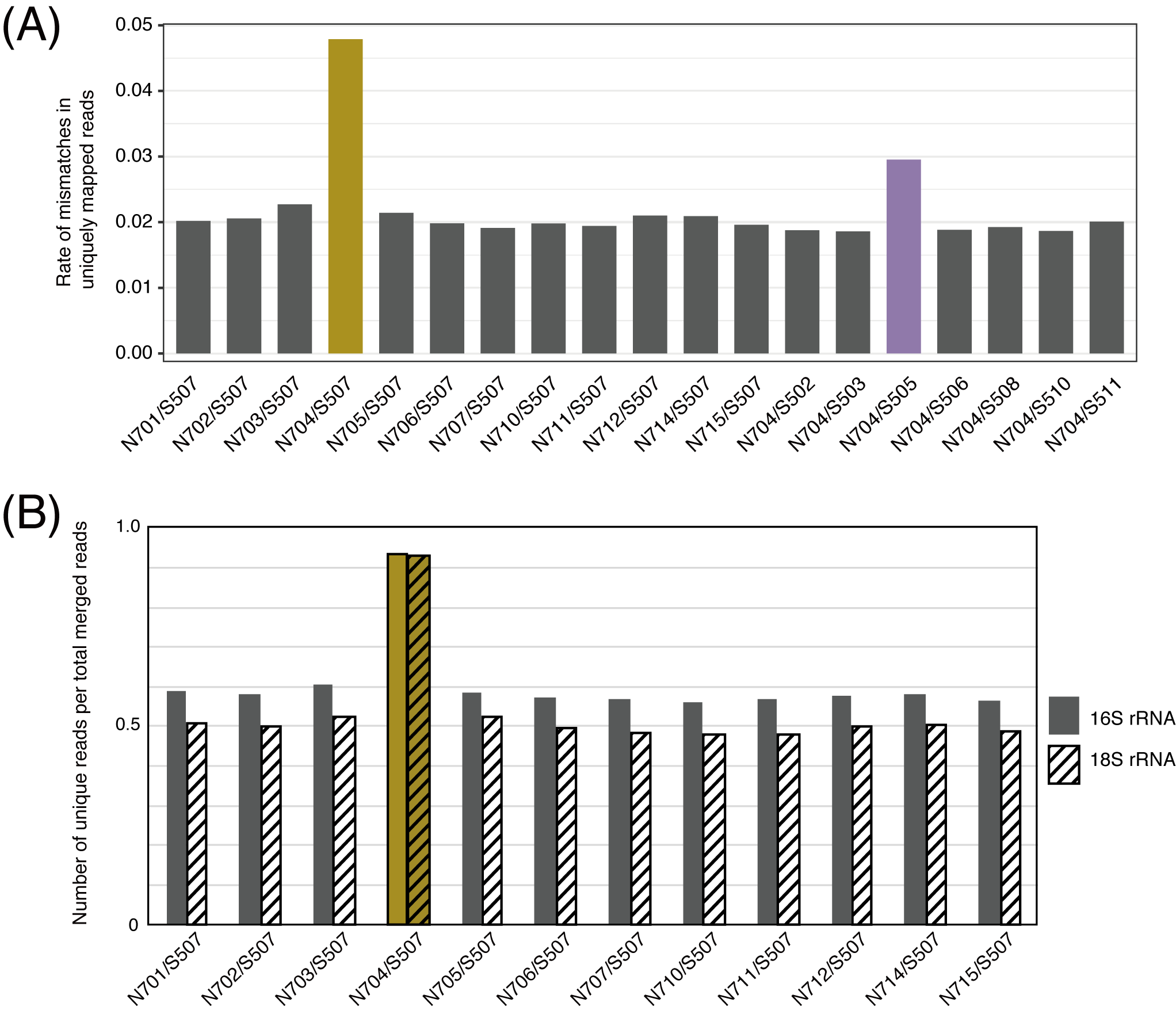

Supplement: dsaa017_Supplementary_Data [file dsaa017_supplementary_data.zip › Fig S3.tif]

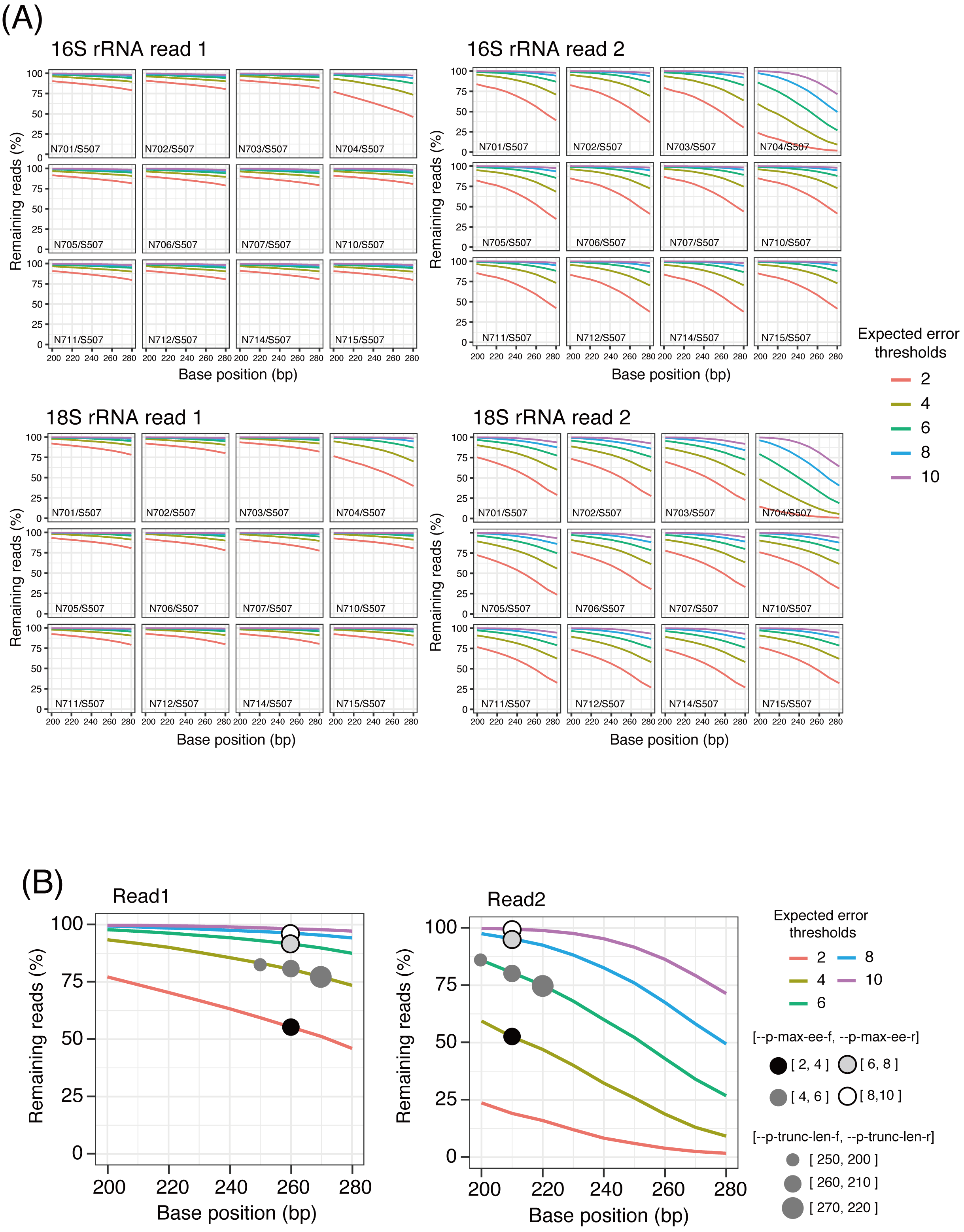

Supplement: dsaa017_Supplementary_Data [file dsaa017_supplementary_data.zip › Fig S4.tif]

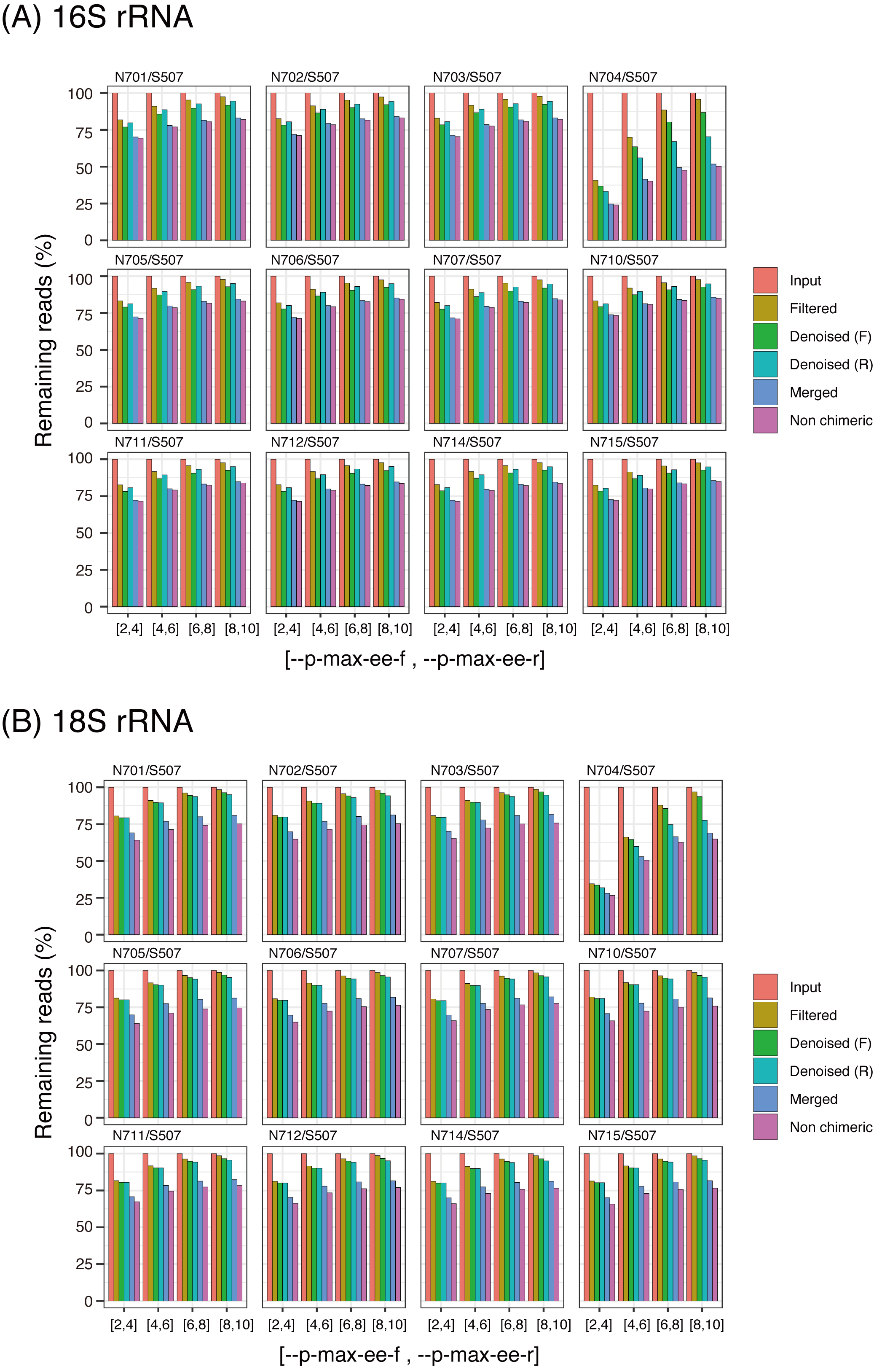

Supplement: dsaa017_Supplementary_Data [file dsaa017_supplementary_data.zip › Fig S5.tif]

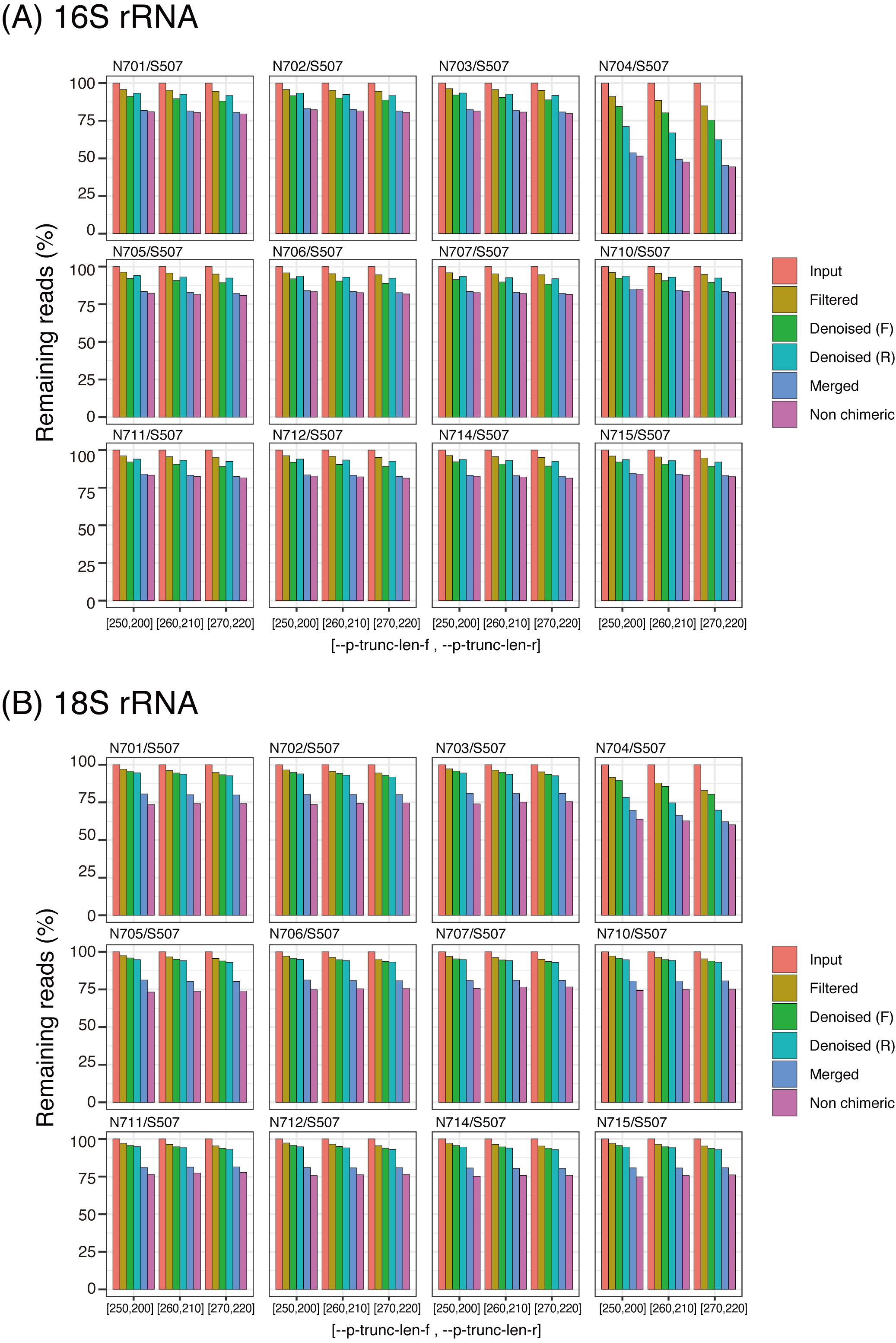

Supplement: dsaa017_Supplementary_Data [file dsaa017_supplementary_data.zip › Fig S6.tif]
